# A Fast Dense Feature-Matching Model for Cross-Track Pushbroom Satellite Imagery

**DOI:** 10.3390/s18124182

**Published:** 2018-11-29

**Authors:** Wen-Liang Du, Xiao-Yi Li, Ben Ye, Xiao-Lin Tian

**Affiliations:** 1 Faculty of Information Technology, Macau University of Science and Technology, Macau, China; du_wenliang@foxmail.com (W.-L.D.); allenleexiao@gmail.com (X.-Y.L.); 2 The Space Science Institute/Lunar and Planetary Science Laboratory, Macau University of Science and Technology, Macau, China; zhyeben@126.com

**Keywords:** fast dense feature-matching, pushbroom satellite imagery, epipolar resampling

## Abstract

Feature-based matching can provide high robust correspondences and it is usually invariant to image scale and rotation. Nevertheless, in remote sensing, the robust feature-matching algorithms often require costly computations for matching dense features extracted from high-resolution satellite images due to that the computational complexity of conventional feature-matching model is $O(N^{2})$. For replacing the conventional feature-matching model, a fast dense (FD) feature-matching model is proposed in this paper. The FD model reduces the computational complexity to linear by splitting the global one-to-one matching into a set of local matchings based on a classic frame-based rectification method. To investigate the possibility of applying the classic frame-based method on cross-track pushbroom images, a feasibility study is given by testing the frame-based method on 2.1 million independent experiments provided by a pushbroom based feature-correspondences simulation platform. Moreover, to improve the stability of the frame-based method, a correspondence-direction-constraint algorithm is proposed for providing the most favourable seed-matches/control-points. The performances of the FD and the conventional models are evaluated on both an automatic feature-matching evaluation platform and real satellite images. The evaluation results show that, for the feature-matching algorithms which have high computational complexity, their running time for matching dense features reduces from hours level to minutes level when they are operated on the FD model. Meanwhile, based the FD method, feature-matching algorithms can achieve comparable matching results as they achieved based on the conventional model.

## 1. Introduction

Feature-based matching is highly robust to image identity changes and distortions, which makes it provide high confident correspondences [1]. Therefore, in remote sensing (RS), feature-based matching is widely used in applications with complex image changes such as wide-baseline stereo matching [2,3,4] and bundle adjustment [5,6]. Furthermore, the dense and redundancy matched features in satellite imagery can be used for reconstructing high-resolution Digital Terrain Models (DTM) [3,7,8], constructing a constraint initializer for the next step of dense matching [9] and estimating Exterior Orientation/georeferencing Parameters (EOP) [10] which may be not provided for commercially available [11].

However, due to that the feature-matching algorithms usually require costly computations, they could be barely used for matching all features extracted from high-resolution pushbroom satellite imagery. For instance, a graph based method Graph Transformation Matching (GTM) [12] was introduced to enforce the spatial constraint by constructing a median K-NN graph and its time computational complexity is $O(N^{3})$. Based on the basic idea of GTM, some algorithms were proposed for improving the robustness and accuracy of it: instead of using a fixed average distance for each image, the Weight Graph Transformation Matching (WGTM) algorithm [13] used the angular distance between edges, which connect a feature-point to its K-NN graph, as the weight; the Spatial Order Constraints Bilateral-Neighbor Vote (SOCBV) [14] algorithm replaced the undirected K-NN graph with a directed one and formulates the feature-matching problem as a binary discrimination problem; the Spatial Order Constraints (RSOC) [15] formulated the feature-matching as an optimization problem for considering both local structure and global information; the Neighborhood Spatial Consistent Matching (NSCM) [16] algorithm was proposed to remove outliers whose offsets between matched features have sudden mutations. The computational complexities of WGTM, SOCBV, RSOC and NSCM are $O(N^{2}log\left(N\right))$, $O(N^{2}log\left(N\right))$, $O(N^{3})$ and $O(N^{2})$ respectively. Hence, unfortunately, the aforementioned algorithms could not be applied on matching dense features extracted from high-resolution satellite imagery, since the numbers of dense features are usually over 10,000 and the aforementioned algorithms will cost days or even run out of memory for matching the dense features [17].

Moreover, the existing feature-matching algorithms are mostly based on the conventional feature-matching model—i.e., before they removing false-matches, the putative matches have to be generated by a global one-to-one matching whose computational complexity is $O(N^{2})$. Therefore, even some feature-matching algorithms (the locally linear transforming (LLT) [18] and a probabilistic method [19]) can reduce the computational complexity of removing false-matches to linearithmic, their computational complexities of generating putative matches are still $O(N^{2})$.

For replacing the conventional feature-matching model, this paper presents a fast dense (FD) feature-matching model which splits the global one-to-one matching into numerous dynamic local matchings so that the complexity of generating putative matches is reduced to linear. Moreover, based on the FD model, feature-matching algorithms with high computational complexity could also be used for matching dense features extracted from high-resolution satellite imagery since the numbers of features in each local matching are much less than the original number of features in the global matching. Note that, the definition of dense feature-matching in this paper is given as—*matching images with features as dense as possible*. Hence, the goal of dense feature-matching is to match the high-resolution images with all the extracted dense features.

In particular, the FD model is designed to match features from cross-track pushbroom satellite imagery. Unlike frame cameras that have well-known epipolar geometry, the physical structures of linear pushbroom cameras are more diverse and complex; the resulting epipolar curves of linear pushbroom cameras are hyperbola-shaped not straight [20,21]. As a result, it is almost impossible to define the epipolar geometry of linear pushbroom cameras rigorously [22].

Nevertheless, the FD model only aims to segment the global matching not eliminating the vertical-parallax of stereo images. Consequently, there is no need to apply the rigorous resampling method on the FD model. In this paper, the process of minimizing the vertical-parallaxes of the rectified correspondences is solved by a classic frame-based rectification method [23] which is well-established and easily-implemented. To investigate the possibility of applying the classic frame based method on cross-track pushbroom satellite imagery, a comprehensive feasibility study is given in this paper. Based on this study, the larger difference of emission angles the satellite cameras have, the more complicated epipolar geometry they will get. As a result, the frame-based method may not work in pushbroom images which have large difference of emission angles. For increasing the success rate of the frame-based method on cross-track satellite imagery, a Correspondence-Direction-Constraint (CDC) algorithm is proposed in the FD model for obtaining the most favourable seed-matches.

To evaluate the FD method, a cross-track pushbroom based automatic feature-matching evaluation platform [17] is introduced for providing comprehensive experiments end evaluating the matching results automatically. Meanwhile, 22 pairs of real cross-track pushbroom satellite images were also prepared for both training (2 pairs) the FD model and testing (20 pairs) the FD and the conventional models.

The primary research contributions of the FD model can be summarized as follows:

1. Based on the FD model, the computational complexity of generating putative feature-correspondences is reduced from $O(N^{2})$ to linear.
2. Features in a certain local matching are independent to the features in other local matchings. Hence, feature-matching can be operated in parallel by applying the FD model.
3. A comprehensive feasibility study of applying the classic frame-based rectification method on cross-track pushbroom satellite imagery is given in this paper.
4. Based on the CDC algorithm, the success rate of minimizing vertical-parallaxes of the pushbroom stereo images by the frame-based method is improved significantly.

The rest of this paper is organized as follows: the feasibility study of applying the classic frame-based method [23] on cross-track pushbroom satellite imagery is introduced in Section 2. The implementation detail of the FD model is described in Section 3. Parameters setting and the performance evaluation of the FD model are discussed in Section 4. Finally, the paper is concluded in Section 5.

## 2. Feasibility Study

Due to that the epipolar geometries of frame cameras and cross-track linear pushbroom cameras are different, a feasibility study of applying the classic frame-based rectification method [23] on cross-track pushbroom stereo images is given in this section. It includes (1) introducing the epipolar geometry of linear pushbroom cameras according to [21]; (2) contrasting the differences of the original and rectified epipolar curves projected by cross-track pushbroom geometry and the original and rectified epipolar lines calculated by the frame-based rectification method; (3) investigating the stability of the frame-based method on a large number datasets provided by a cross-track pushbroom based automatic feature-matching evaluation platform [17].

### 2.1. Epipolar Geometry of Linear Pushbroom Cameras

For a point **a** $=\left(a,b,c\right)$ in the world plane, it is viewed by two linear pushbroom cameras with camera matrices *E* and $E^{\prime }$. The points in the image planes according to the point a are **x** $={\left(x,y\right)}^{T}$ and $x^{\prime }={\left(x^{\prime },y^{\prime }\right)}^{T}$. These three points satisfy the following equations:

(1) $$\left[\begin{array}{c} x\\ wy\\ w \end{array}\right]=E\left[\begin{array}{c} a\\ b\\ c\\ 1 \end{array}\right],\left[\begin{array}{c} x^{\prime }\\ w^{\prime }y^{\prime }\\ w^{\prime } \end{array}\right]=E^{\prime }\left[\begin{array}{c} a^{\prime }\\ b^{\prime }\\ c^{\prime }\\ 1 \end{array}\right],$$

where the *w* and $w^{\prime }$ are scale factors and these two equations can be rewritten as:

(2) $$\left[\begin{array}{cccccc} e_{11} & e_{12} & e_{13} & e_{14}-x & 0 & 0\\ e_{21} & e_{22} & e_{23} & e_{24} & y & 0\\ e_{31} & e_{32} & e_{33} & e_{34} & 1 & 0\\ e{}_{11}^{\prime } & e{}_{12}^{\prime } & e{}_{13}^{\prime } & e{}_{14}^{\prime }-u^{\prime } & 0 & 0\\ e{}_{21}^{\prime } & e{}_{22}^{\prime } & e{}_{23}^{\prime } & e{}_{24}^{\prime } & 0 & y^{\prime }\\ e{}_{31}^{\prime } & e{}_{32}^{\prime } & e{}_{33}^{\prime } & e{}_{34}^{\prime } & 0 & 0 \end{array}\right]\left[\begin{array}{c} a\\ b\\ c\\ 1\\ -w\\ -w^{\prime } \end{array}\right]=0.$$

The 6×6 matrix in Equation (2) can be denoted as $A\left(E,E^{\prime }\right)$ such that the Equation (2) is a set of linear equations. If this system have a solution, then det $A\left(E,E^{\prime }\right)=0$. This rises to a cubic equation $g\left[\begin{array}{cccc} x & y & x^{\prime } & y^{\prime } \end{array}\right]=0$, where the coefficients of *g* are determined by the entries of *E* and $E^{\prime }$. Since there are no terms of $x^{2}$, ${x^{\prime }}^{2}$, $y^{2}$ and $y^{2}$ in Equation (2), there exists a matrix *F* satisfied $g\left[\begin{array}{cccc} x & y & x^{\prime } & y^{\prime } \end{array}\right]=0$ and can be written as:

(3) $$\left[\begin{array}{cccc} x^{\prime } & x^{\prime }y^{\prime } & y^{\prime } & 1 \end{array}\right]F\left[\begin{array}{c} x\\ xy\\ y\\ 1 \end{array}\right]=0.$$

The matrix *F* in the above equation is the fundamental matrix of linear pushbroom cameras. It must be satisfied by any pair of corresponding image points ${\left(x,y\right)}^{T}$ and ${\left(x^{\prime },y^{\prime }\right)}^{T}$. Now, considering the locus of all possible matched points ${\left(x^{\prime },y^{\prime }\right)}^{T}$ and writing the ${\left(x^{\prime },y^{\prime }\right)}^{T}$ as ${\left(\alpha ,\beta ,\gamma ,\delta \right)}^{T}$ in Equation (2). Then a equation of a hyperbola-epipolar loci could be obtained: $\alpha x^{\prime }+\beta x^{\prime }y^{\prime }+\gamma y^{\prime }+\delta =0$. In other words, unlike the frame cameras whose epipolar lines are straight lines, the epipolar lines of linear pushbroom cameras are hyperbolas. The difference between the epipolar lines of the two kinds of cameras are simulated in the following section.

### 2.2. Contrast of Epipolar Curves and Lines

**Figure 1:**
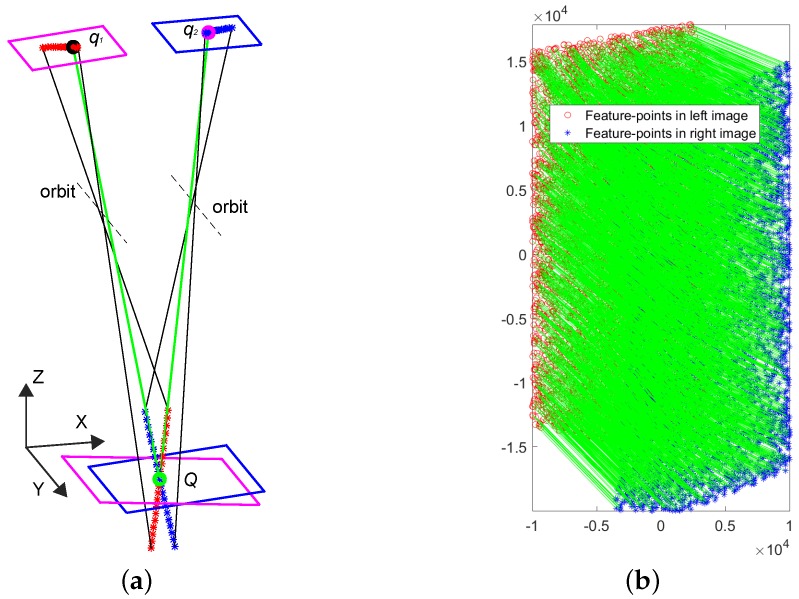
Pushbroom satellite stereo systems simulation. (**a**) An example of a cross-track linear pushbroom satellite stereo imaging system. (**b**) 5000 correspondences simulated by HiRISE imaging system.

An example of projecting epipolar curves of a cross-track pushbroom satellite camera model is illustrated in Figure 1a, where the magenta and blue parallelograms at the top are the image planes of two linear pushbroom cameras, the magenta and blue parallelograms at the bottom are the world planes of the two cameras. The black and magenta cycles in image planes, which are $q_{1}$ and $q_{2}$, are two conjugate points that projected by the object point *Q* (which is the green cycle in the world plane), where $q_{1}$, $q_{2}$ and *Q* construct an epipolar plane. Then some evenly-distributed points are selected along the lines of $Qq_{1}$ and $Qq_{2}$; epipolar curves are obtained by project evenly-distributed points to image planes (red and blue stars in image planes of Figure 1a). It is obviously that the epipolar curves shown in Figure 1a are not straight lines.

**Figure 2:**
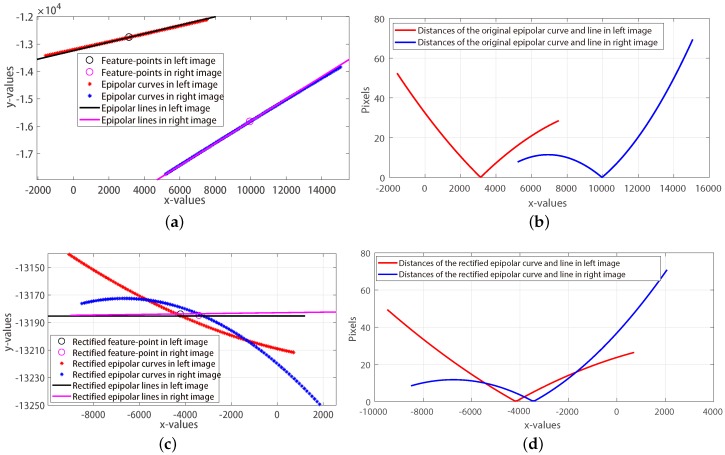
The most different original and rectified epipolar curves and lines. (**a**,**b**) The original epipolar curves and lines and their corresponding distances. (**c**,**d**) The rectified epipolar curves and lines and their corresponding distances.

To compare the epipolar curves and lines, the pushbroom camera (shown in Figure 1a) is simulated as HiRISE (High Resolution Imaging Science Experiment cameras which is on board NASA’s Mars Reconnaissance Orbiter (MRO)) camera and 5000 pairs of epipolar curves are generated by the cross-track pushbroom based platform [17]; The corresponding epipolar lines are calculated by the frame-based method [23]. More specifically, the slewing and emission angles of the simulated cameras can be randomly rotated in $-30^{\circ }$ to $30^{\circ }$ and the differences of slewing and emission angles of them are set as $15^{\circ }$. Figure 1b shows an example of 5000 pairs of simulated correspondences and Figure 2a shows the most different pair of epipolar curves and lines among the 5000 simulated correspondences, where the black and magenta lines are the epipolar lines calculated by the frame-based rectification method. At first glance of Figure 2a, the epipolar curves and lines are looks like that they are arranged together. However, the more specific distances of this pair of epipolar curves and lines (in Figure 2b) tells us that not only the epipolar curves and lines are not arranged in straight lines but also the epipolar curves are not straight lines.

Then, this pair of epipolar curves and lines are rectified by the classic frame-based method. The rectified results and the distance of rectified curves and lines are shown in Figure 2c,d. As can be seen, if the searching range could be limited in a reasonable value, the feature-correspondences could be segmented in a set of local matchings so that the computational complexity of feature-matching could be reduced.

### 2.3. Investigating the Stability of the Classic Frame-Based Method

7000 datasets were simulated based on the cross-track pushbroom based feature-matching evaluation platform [17]; the specification of the platform was set as the HiRISE camera. The datasets were classified in 7 groups by various differences of slewing and emission angles, and each group contained 1000 datasets. Moreover, each dataset had 5000 feature-correspondences which are all true-matches. Then, for each dataset, the frame-based method was performed based on sampling 10, 250 and 500 random evenly-distributed seed-matches/control-points; the method was repeated in 1000 times for each sampling. Therefore, totally, 2.1 million independent experiments were prepared.

The success rate can be described as Equations (4) and (5) which are calculated based on the *j*-th experiment. In these equations, $T_{j}$ indicates a threshold that restricts the valid differences of the absolute value of y-disparities of the rectified features, $Y_{j}$ is the set of y-values of the original features in the left image, *L* represents that how many matching areas we want to split from the global matching (in this paper *L* is set as 250 which means there are about 20 correspondences in each local matching, since each dataset has 5000 correspondences), *N* represents the number of correspondences, ${\hat{y}}_{i}$ and ${\hat{y}}_{i}^{\prime }$ are the y-values of *i*-th rectified features in the left and right images respectively, $sr_{j}$ represents that whether the rectification method could get a reasonable result in this experiment. In other words, for a certain experiment, the rectified method succeeds if the number of qualified rectified correspondences is over 99%.

(4) $$T_{j}=\frac{\max\left(Y_{j}\right)-\min\left(Y_{j}\right)}{L},$$

(5) $$sr_{j}=\left\{\begin{array}{lll} 1 & if & \frac{\sum_{i=1}^{N}\mathrm{bool}\left(\left|{\hat{y}}_{i}-{{\hat{y}}^{\prime }}_{i}\right|\geq T_{j}\right)}{N}\geq 0.99\\ 0 & else & \end{array}\right.,$$

(6) $$SR_{k}=\frac{\sum_{j=1}^{M}sr_{j}}{M}.$$

**Figure 3:**
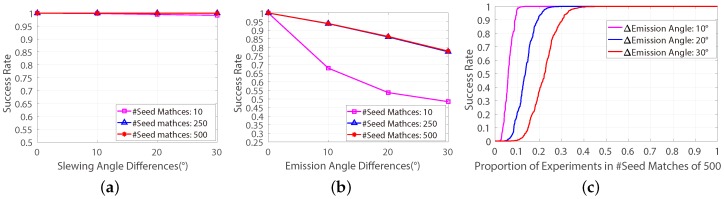
The success rates of the rectification method performed on the 7000 simulated datasets. (**a**) The success rates on datasets with various differences of slewing angles. (**b**) The success rates on datasets withe various differences of emission angles. (**c**) The success rates on datasets with 500 seed-matches and various differences of emission angles.

Figure 3a,b show the success rates of the rectification method on datasets with various differences of slewing and emission angles. The success rates for each classes are calculated as the average value of $SR_{k}$ in Equation (6), where *M* represents the number of repeating time of the rectification method and $SR_{k}$ represents the success rate in the *k*-th dataset (7000 datasets in total). It is obviously that, the difference of slewing angles doesn’t affect the success rate, whereas the success rate declines significantly while increasing the difference of emission angles, and the more seed-matches are selected the higher success rate could be achieved by the rectification method. But the improvement will decline while increasing the number of seed-matches. Moreover, as can be seen in Figure 3c, for the datasets with the difference of emission angles of $30^{\circ }$ and the number of seed-matches of 500, there are almost 10% of them that the method never succeed for even just one time over the 1000 repeated independent experiments, even if their average success rate is over 75% (see in Figure 3d). Therefore, it may be like trying to find a needle in a haystack that directly applying the classic frame-based rectification method on some extreme cases of the pushbroom satellite imagery.

**Figure 4:**
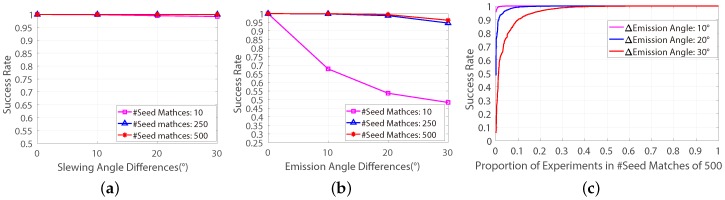
The success rates of the rectification method performed on the 7,000 simulated datasets based on the CDC algorithm. (**a**) The success rates on datasets with various differences of slewing angles. (**b**) The success rates on datasets withe various differences of emission angles. (**c**) The success rates on datasets with 500 seed-matches and various differences of emission angles.

Therefore, one of the objectives of the proposed FD model is to increase the success rate of applying the well-established and classic frame-based rectification method on pushbroom satellite imagery. In the FD model, a Correspondence-Direction-Constraint (CDC) algorithm is proposed to obtain the most favourable seed-matches for the frame-based method. Figure 4 shows the success rates of the rectification method based on the CDC algorithm. According to Figure 4, the success rates of the frame-based rectification method is improved significantly when it is based on the FD model. Moreover, the frame-based rectification method can be used for getting reasonable splitting results in all datasets and, for almost 99% datasets with the difference of emission angle of $30^{\circ }$, the success rate of rectification method based on the FD model is over 50% (see Figure 4c).

## 3. The Fast Dense Feature-Matching Model

The most different of the conventional feature-matching model and the proposed FD model is that the proposed model splits the global one-to-one feature-matching into numerous of local one-to-one matchings. But they are both involved in feature extraction and generating putative correspondences.

### 3.1. Feature Extraction

In feature-based matching, features could be selected by the significant regions (lakes, fields), lines (region boundaries, coastlines, rivers), points (region corners, descriptors) [24]. Owing to the rapid development of robust and efficient descriptors (e.g., Scale-Invariant Fourier Transform (SIFT) [25] and Speeded-Up Robust Features (SURF) [26]), points are the most popular features for feature-based image registration in this decade. Among all the points’ features, SIFT is the most commonly used one because of its high robustness and invariant to image scale and rotation.

In this paper, SIFT is adopted to extract robust features and is implemented by the open-source VLFeat toolbox [27]. Examples of a pair of Martian stereo images (captured by HiRISE) and their feature-points extracted by SIFT are shown in Figure 6a,b. Obviously, SIFT could give very dense features (21,062 and 18,179 feature-points are extracted from the left and right images).

### 3.2. Global Matching Splitting

**Figure 5:**
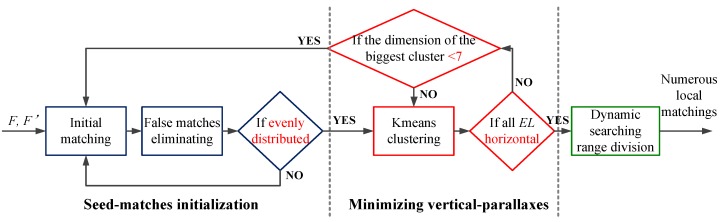
The flow chart of global matching splitting.

The flowchart of this procedure is shown in Figure 5. Firstly, the initial evenly-distributed seed-matches should be obtained. Then the vertical-parallaxes of the seed-matches are minimized by the correspondence-direction-constraint (CDC) algorithm and the classic frame-based rectification method. Eventually, the numerous searching ranges are dynamically divided based on the locations of the initial evenly-distributed seed-matches.

#### 3.2.1. Seed-Matches Initialization

**Figure 6:**
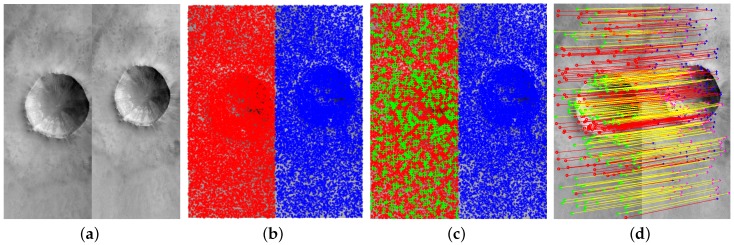
An example of the seed-matching initialization. (**a**) A pair of Martian stereo images captured by HiRISE. (**b**) Features-points extracted by SIFT operator (red and blue stars denote *U* and $U^{\prime }$ respectively). (**c**) Feature sampling from *U* (green crosses represent *u*). (**d**) False-matches elimination by RANSAC (red cycles, blue crosses and red lines represent the eliminated false-matches; blue cycles, magenta crosses and yellow lines represent the initial seed-matches).

For the present, suppose we have a pair of stereo images (see in Figure 6a), and they are denoted as *I* and $I^{\prime }$ respectively. Their features are extracted by SIFT operator and denoted as *U* and $U^{\prime }$ (see in Figure 6b). The initial putative correspondences are generated by matching $U^{\prime }$ and *n* sampled features randomly sampled from *U*, where the *n* features are denoted as *u* (see in Figure 6c). Then, the false-matches of the initial putative correspondences are eliminated by the Random Sample Consensus algorithm (RANSAC, see Figure 6d) and the resulting true-matches construct the initial seed-matches whose corresponding feature-points in the left and right images are denoted as In and $In^{\prime }$ respectively.

The locations of the initial seed-matches are used for adjusting the searching ranges of the FD model dynamically. Hence, the initial seed-matches are expected to be evenly-distributed. For ensuring the initial seed-matches are evenly-distributed, Hypothesis 1 is proposed in this paper.

**Hypothesis** **1.** *Given p evenly-distributed points P, and sample q evenly-distributed points Q from P. Construct Delaunay graphs of P and Q as DG(P) and DG(Q). Denote the averages of lengths of the edges in DG(P) and DG(Q) as LP and LQ. The proportion of LQ to LP, which is denoted as $R_{QP}$, should satisfy the following equation:*

(7) $$R_{QP}=\frac{LQ}{LP}\geq \alpha \sqrt{\frac{p}{q}},$$

where α is the adjustment factor (0≤α≤1), and $\sqrt{\frac{p}{q}}$ is the ideal proportion.

**Figure 7:**
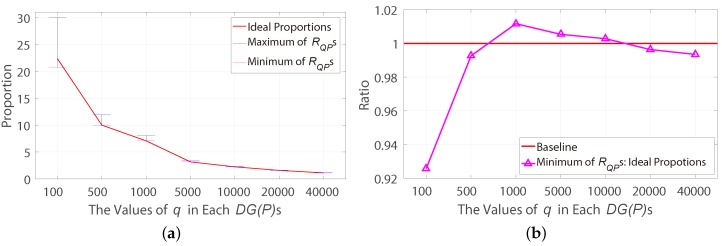
The verified results of the Hypothesis 1. (**a**) The proportion of the maximum and minimum $R_{QP}$s and ideal proportions. (**b**) The ratios of the minimum $R_{QP}$s to their corresponding ideal proportions.

Hypothesis 1 is verified by 7 million independent experiments which contain 1000 independent DG(P)s with 50,000 evenly-distributed points, and 100, 500, 1000, 5000, 10,000, 20,000, 40,000 points are randomly sampled from each DG(P) in 1000 times to construct the DG(Q)s. The resulting proportion of the maximum and minimum $R_{QP}$ and ideal proportions in various sampling numbers is shown in Figure 7a. In these experiments, there are more than 99% $R_{QP}$s larger than or equal to the corresponding ideal proportions. The more specific results of ratios of the minimum $R_{QP}$s to their corresponding ideal proportions are shown in Figure 7b, which proves that the ideal proportion is the good indicator of whether the seed-matches are evenly-distributed. Moreover, for some images that the seed-matches cannot be evenly-distributed (e.g., images with many flat areas), a lower value of the adjustment factor α could be set.

In practice, DG(P) and DG(Q) are constructed by *u* and In respectively. If they are not satisfied the Equation (7), the procedure of initializing the seed-matches will start over.

#### 3.2.2. Minimizing the Vertical-Parallaxes of Seed-Matches

The vertical-parallaxes of seed-matches is minimized by introducing the classic rectification method [23]. In order to increase the success rate of the classic method, a correspondence-direction- constraint (CDC) algorithm is proposed for identifying the most favourable combination of the initial seed-matches.

(8) $$V=In-In^{\prime }.$$

(9) $$\sum_{i=1}^{n}\mathrm{bool}\left(S_{i}\leq 0.01\right)=n.$$

The combinations of the initial seed-matches are generated by the k-means [28] clustering method. Firstly, the vectors of the initial seed-matches (denoted as *V*) are calculated by Equation (8). Then, for the *k*-th iteration of the CDC, the seed-matches are classified in *k* combinations by k-means method according to the slopes of *V*. More specifically, the classify precedure is done in the line 3 of Algorithm 1, where “directions” means that classify the *V* by their directions. For each iterations, the epipolar lines of *u* (denoted as EL) are calculated by the classic method based on the corresponding combinations. For a certain combination, if all EL are horizontal (the “horizontal” is defined as Equation (9), where $S_{i}$ is a slope of epipolar line in EL and *n* is the number of *u*), the CDC algorithm will stop and output the optimal EL ($\hat{EL}$) and the projective transformation matrices (*H* and $H^{\prime }$). The rectified In and $In^{\prime }$ are denoted as $\hat{In}$ and $\hat{In^{\prime }}$ and calculated as:

(10) $$\hat{In}=HIn,$$

(11) $$\hat{In^{\prime }}=H^{\prime }In^{\prime }.$$

An example of the results of rectification is shown in Figure 9b. As can be seen, the y-disparities of the rectified seed-matches are very small. A simple pseudocode is given in Algorithm 1. In particular, in the line 4 of Algorithm 1, the algorithm will stop and the procedure of initializing seed-matches will start over when the maximum number of the combinations is less than 7, since 7 pairs of correspondences at least are needed for the classic rectification method [23].

**Algorithm 1** The Correspondence-Direction-Constraint (CDC) Algorithm.**Input:**In and $In^{\prime }$: feature-points of the initial seed-matches in the left and right images respectively; *u*: *n* feature-points randomly sampled from *U*; *V*: vectors of the initial seed-matches.**Output:**$\hat{EL}$: the optimal EL; *H* and $H^{\prime }$: projective transformation matrices. 1: GoodComb = False; *k* = 1;2: **while**GoodComb==False**do**3:  Combs=kmeans(*V*, *k*, directions);4:  **if** the dimension of the biggest cluster less than 7 **then**5:   break6:  **end if**7:  **for** i=1:k **do**8:   [EL,*H*,$H^{\prime }$]=RectificationMethod($In\left(Combs_{i}\right)$, $In^{\prime }\left(Combs_{i}\right)$,*u*);9:   **if** all EL are horizontal **then**10:    GoodComb = True;11:    $\hat{EL}$=EL;12:    break13:   **end if**14:  **end for**15: **end while**

#### 3.2.3. Dynamic Searching Ranges Division

The rectified feature-points of the left and right images are obtained based on Equations (12) and (13) and defined as $\hat{U}$ and $\hat{U^{\prime }}$. A searching line is set to traverse from the top to the bottom of the $\hat{U}$ and $\hat{U^{\prime }}$, meanwhile the searching ranges are divided in both left and right images. The coverage of the searching range in left image is denoted as *T* and defined as Equation (14), where $X_{\max}$ and $X_{\min}$ are the maximum and minimum columns of $\hat{U}$, $\hat{S}$ is the absolute maximum slope in $\hat{EL}$. On the other hand, in order not to omit any potential true-matches, the coverage of the searching range in the right image is two times larger than *T*.

(12) $$\hat{U}=HU,$$

(13) $$\hat{U^{\prime }}=H^{\prime }U^{\prime }.$$

(14) $$T=2\left|\left(X_{\max}-X_{\min}\right)\hat{S}\right|$$

**Figure 8:**
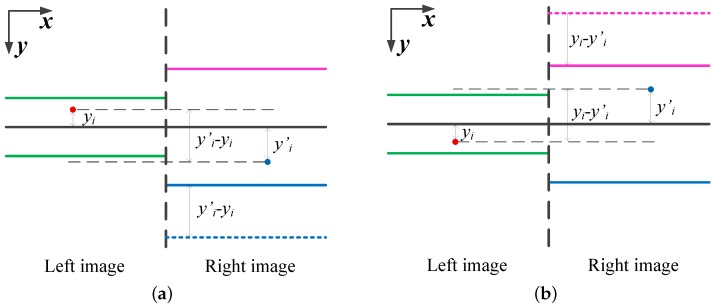
Two cases of adjusting the *i*-th searching range (black lines represent the searching line; the area between green lines are the searching range in the left image (*T*); blue and magenta lines represent the original top and bottom of the searching range in the right images (2T); blue and magenta dashed lines are the updated top and bottom of the searching range). (**a**) Updating the top of the searching range. (**b**) Updating the bottom of the searching range.

**Figure 9:**
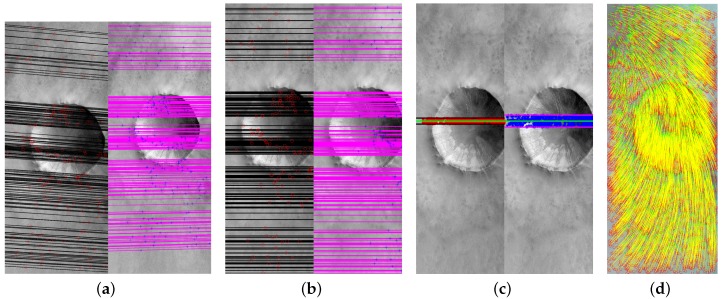
An example of minimizing the vertical-parallaxes of seed-matches, searching ranges and the dense matching result based on.the FD model. (**a**,**b**) the original and rectified epipolar lines of the initial seed-matches (black and magenta lines epipolar lines in the left and right images; red cyles and blue crosses in (**a**) are In and $In^{\prime }$, in (**b**) are $\hat{In}$ and $\hat{In^{\prime }}$). (**c**) An example of a local matching (the green line is the searching line, the area between black lines and the area between magenta lines represent a certain searching area). (**d**) An example of dense feature-matching results (7247 matched correspondences in this case; red cycles, blue crosses and yellow lines represent the resulting correspondences of *U* and $U^{\prime }$).

As can be seen in Figure 9b, the rectified epipolar lines are not exactly linked up to each other. Hence, the searching ranges in the right images should be dynamically adjusted so that most of the potential true-matched could be covered. The left and right images share the same searching lines, but the top and the bottom of each searching ranges in the right image should be dynamically adjusted according to the locations of the nearest seed-matches of the corresponding searching lines. More specifically, for the *i*-th searching searching range, if its nearest right seed-match’s y-value is larger than the left one’s, the top of the searching range in the right image should be extended as Equation (15) (see Figure 8a). Otherwise, the bottom of the searching range in the right image should be extended as Equation (16) (see Figure 8b).

(15) $$TOP_{i}=TOP_{i}+\left({y_{i}}^{\prime }-y_{i}\right)\left[\mathrm{bool}\left(y_{i}<{y_{i}}^{\prime }\right)\right]$$

(16) $$BOTTOM_{i}=BOTTOM_{i}-\left(y_{i}-{y_{i}}^{\prime }\right)\left[\mathrm{bool}\left(y_{i}\geq {y_{i}}^{\prime }\right)\right]$$

### 3.3. Generating Putative-Correspondences

In this procedure, a set of putative-correspondences are generated by matching the features from each searching ranges in left and right images. Then, the false-matches in each set of putative-correspondences could be eliminated by the existing feature-matching algorithms. An example of features in a searching range is shown in Figure 9c. As can be seen, matching features in this searching range is much more easier than matching features in all the image.

Moreover, the features in each searching ranges are independent to each other. Therefore, this procedure could be operated in parallel. Figure 9d shows a matching result by the WGTM algorithm based on the FD model. As can be seen, preserved correspondences are very dense.

## 4. Experimental Results

As mentioned before, the main purpose of this paper is to reduce the computational complexity of feature-matching by replacing the conventional feature-matching model with the proposed FD model. Therefore, the performances of the FD model and the conventional feature-matching model were evaluated by performing three state-of-art feature-matching algorithms (LLT [18], SOCBV [14] and WGTM [13]) on both an automatic feature-matching evaluation platform [17] and 22 pairs of real HiRISE stereo images based on these two models (in Section 4.4 and Section 4.5). The computational complexity of the proposed FD model, the datasets and the parameters mentioned previously are discussed in Section 4.1, Section 4.2 and Section 4.3 respectively.

In addition, all the experiments are performed on two personal computers, each with an Intel i5 CPU (3.3 GHz and 4 cores) and 16-GB RAM. The program of the proposed FD model is wrote in MATLAB code, where the source codes are available at https://github.com/WenliangDu/FastDenseFeatureMatchingModel.

### 4.1. Computational Complexity

If the FD model is not operated in parallel, its time complexity is $O\left({\left(\frac{N}{k}\right)}^{2}\times k\right)$ which can be simplified as O(aN), where *N* denotes the total number of feature-correspondences, *a* denotes the average number of feature-correspondences in each local matchings ($a=\frac{N}{k}$).

The worst case of the FD model is that there is only one local matching in the FD model, which means a=N and the worst complexity of the FD model is $O(N^{2})$. The best case is that each correspondences are exactly arranged in each independent local matchings so that a=1 and the best complexity of the FD model is O(N). To bring in the best performances of the existing feature-matching algorithms, in the experiments of real HiRISE images, the value of *a* was kept just larger than 300 which is also greatly less than *N* (usually more than 10,000).

### 4.2. Datasets

The datasets provided by the automatic feature-matching evaluation platform [17] can evaluate the performances of the two models automatically. The platform was simulated as the HiRISE camera which is on board NASA’s Mars Reconnaissance Orbiter (MRO). Therefore, the specification of the simulated remote sensor was set as [29]—focal length: 12 m, camera distance: 300 km, field of view (FOV): $1.14{}^{\circ }$, the number of pixels in the linear CCD array: 20,048 (10 RED CCDs), the number of along-track lines: 40,000. The source codes of the platform are available at https://github.com/WenliangDu/RS-FeatureMatchingEvaluatingPlatform.

20 pairs of real HiRISE stereo images were used for testing the two models and the results were evaluated manually. 2 pairs of real HiRISE stereo images were used for training the value of α (in Equation (7)) used in the FD model. The HiRISE stereo images could be downloaded from https://hirise.lpl.arizona.edu.

### 4.3. Parameters Discussion

In Section 3.2, *N* features need to be sampled from *U* and to match all the $U^{\prime }$ for generating the initial seed-matches. In this paper, the value of *N* is set as 1000.

The value of α in Equation (7) is set as 1 for the datasets simulated by the automatic evaluation platform. In contrast, the value of α is trained by the 2 pairs of HiRISE images and set as 0.7, which means the initial seed-matches/control-points generated by RANSAC may be not evenly-distributed, since the features in the flat area may be too similar to each other.

### 4.4. Automatic Performance Evaluation

Four groups of datasets were prepared by the automatic evaluation platform [17] for evaluating the two models in various differences of slewing and emission angles ($0^{\circ }$ to $30^{\circ }$), ratios of false-matches (10% to 50%) and numbers of feature-correspondences (1000 to 5000). For each experiment in all the groups, 50 different datasets were prepared. Hence, the result of each experiment was gathered with the average values of 50 independent tests; totally, 850 datasets were simulated for automatically evaluating.

In the first two groups, the differences of slewing and emission angles were both varied from $0^{\circ }$ to $30^{\circ }$. The ratio of false-matches, which can be also called as outliers’ ratio, was set as 20%. Because the execution time of feature-matching algorithms based on the conventional model is usually to long, the number of feature-correspondences was selected as 2000. Note that, the slewing and emission angles of the platform were randomly sampled from $-30^{\circ }$ to $30^{\circ }$.

The performances of the three state-of-art feature-matching algorithms based on the two matching models were evaluated on four generic criteria [13,30]:

(17) $$Accuracy=\frac{TP+TN}{TP+TN+FN+FP},$$

(18) $$Precision=\frac{TP}{TP+FP},$$

(19) $$Recall=\frac{TP}{TP+FN},$$

(20) $$Specificity=\frac{TN}{TN+FP},$$

where TP and FP denote the number of resulting true-matches and false-matches respectively, FN and TN denote the number of missing true-matches and false-matches. Therefore, the Accuracy describes the degree of true-matches to all matches, the Precision gives the degree of remaining true-matches to all remaining feature-correspondences, the Recall tells the degree of the number of remaining true-matches to the number of all true-matches, the Specificity states the degree of the number of discriminated false-matches to the number of all false-matches.

**Figure 10:**
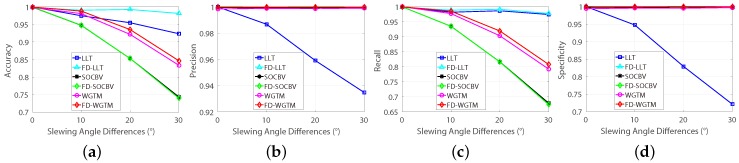
Automatic evaluation results of the three algorithms on datasets with various differences of slewing angles. (**a**) Accuracy. (**b**) Precision. (**c**) Recall. (**d**) Specificity.

The automatic evaluation results of the first group datasets are shown in Figure 10, where the “FD-” means that the corresponding algorithm was performed on the FD model, otherwise the algorithm was performed on the conventional model. Apparently, the abilities of preserving true-matches of SOCBV and WGTM algorithms based on both the two models are declined while increasing the differences of slewing angle (see Figure 10a,c). In contrast, the ability of removing false-matches of the LLT is improved significantly when it is based on the FD method (see Figure 10b,d).

**Figure 11:**
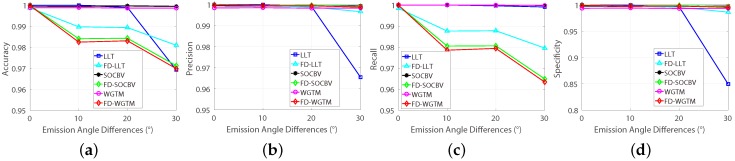
Automatic evaluation results of the three algorithms on datasets with various differences of emission angles. (**a**) Accuracy. (**b**) Precision. (**c**) Recall. (**d**) Specificity.

According to Figure 11, in contrast to the three method based on the conventional model, the abilities of preserving true-matches of the three method based on the FD model are indeed decreased but not insignificant (1 to 0.96, see Figure 11c), which is reasonable, since the epipolar geometry is getting more complicated while increasing the differences of the emission angle. However, when the difference of emission angle goes to 30%, the ability of eliminating false-matches declines of the LLT based on the FD model is greater than it is based on the conventional model.

**Figure 12:**
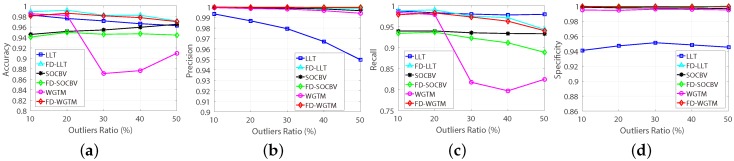
Automatic evaluation results of the three algorithms on datasets with various outliers’ ratio (ratio of false-matches). (**a**) Accuracy. (**b**) Precision. (**c**) Recall. (**d**) Specificity.

The performances of the three matching algorithms on various ratios of false-matches are shown in Figure 12. As can be seen, for higher ratios of false-matches, the ability of WGTM algorithm for preserving true-matches and the ability of LLT algorithm for eliminating false-matches are improved when they are performed on the FD model (see Figure 12c,d). As can be seen in Figure 12d, the three algorithms can remove nearly all the false-matches based on the FD model.

The datasets with various numbers of feature-correspondences were used for evaluating the running time of the two models; the corresponding results are shown in Table 1. Obviously, when SOCBV and WGTM algorithms were performed on the FD model, their operating time reduced substantially then they were performed on the conventional model. Therefore, by applying the FD model, SOCBV and WGTM algorithms could be performed on high-resolution pushbroom satellite images which contain very dense feature-correspondences, even the complexities of them are very high. On the other hand, for the LLT algorithm, its operating time increased slightly when it was performed on the FD model, while its operating time is still very low.

### 4.5. Performance Evaluation on HiRISE images

The three algorithms were only performed on the FD model for matching the 22 pairs of real HiRISE images, because the complexities of SOCBV and WGTM algorithms are so high that they may cost days or even weeks for matching one pair of HiRISE images when they are based on the conventional feature-matching model. Besides, the LLT algorithms runs out of memory for matching some of the 22 pairs of HiRISE images when it is based on the convention model obtained.

**Figure 13:**
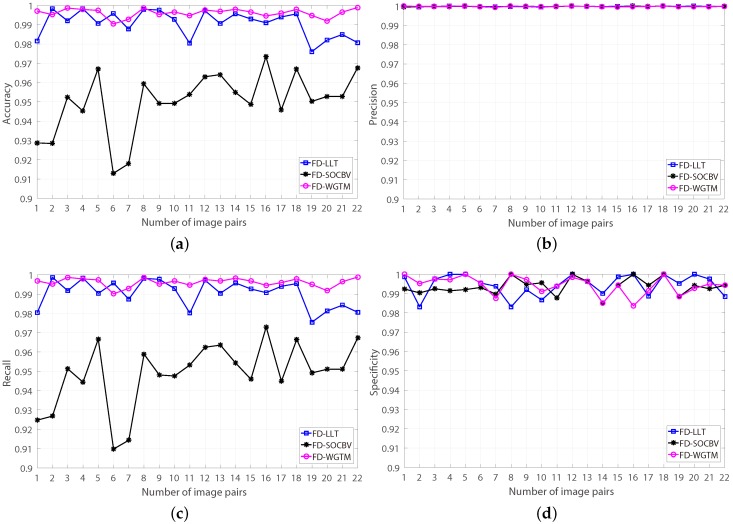
Manual evaluation results of the three algorithms based on FD model for 22 pairs of HiRISE images (the 1 and 2 are training pairs, the rests are testing images pairs). (**a**) Accuracy. (**b**) Precision. (**c**) Recall. (**d**) Specificity.

The results of the three algorithms of matching the 22 pairs of real HiRISE images based on the FD model were evaluated manually. A manual evaluation for a matching result of an algorithm performed on one pair of HiRISE images cost us at least five minutes of human operator time, since the numbers of the resulting correspondences of most matching results are more than 10,000. The evaluating results are shown in Figure 13. It is apparent that, for all the 22 pairs of HiRISE images, all the three algorithms achieved good matching results when they were based on the FD model (more than 98% false-matches can be eliminated).

**Figure 14:**
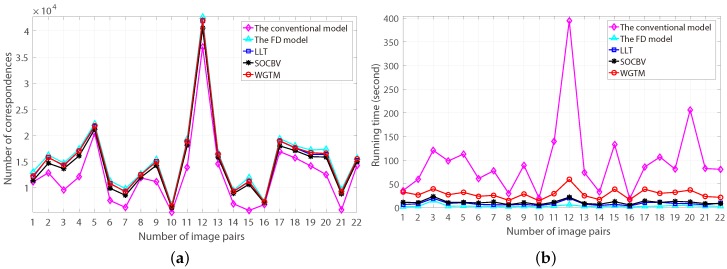
The number of resulting correspondences and running time of the conventional and the FD models and the three algorithms. (**a**) The numbers of putative-correspondences generated by the conventional model and the FD model (magenta and cyan lines) and of the correspondences preserved by the SOCBV, WGTM and LLT algorithms (black, red and blue lines respectively). (**b**) The running time of generating putative-correspondences by the conventional and the FD models (magenta and cyan lines) and of eliminating false-matches by the SOCBV, WGTM and LLT algorithms (black, red and blue lines respectively).

The number of resulting correspondences generated by the three algorithms based on the FD model and the number of putative correspondences generated by the conventional and the FD model are shown in Figure 14a. As can be seen, all the three algorithms preserved more correspondences than the putative correspondences generated by the conventional model, since the FD model obtained much more putative correspondences than the conventional model.

The running time of eliminating false-matching by the three algorithms and generating putative-correspondences by the two models is shown in Figure 14b. Obviously, the running time of the three algorithms and the FD model is all shorter than the running time of the conventional model. More specifically, based on the FD model, the running time of the SOCBV and WGTM algorithms for eliminating false-matches from the 22 pairs of high-resolution HiRISE images is always lower than 1 min, even the computational complexities of the two algorithms are both $O\left(N^{2}\log\left(N\right)\right)$.

## 5. Conclusions

To match dense features extracted from high-resolution cross-track pushbroom satellite imagery in efficiently, this paper proposed a fast dense (FD) feature-matching model. The FD model splits the global one-to-one matching into a large number of local matchings by minimizing the vertical-parallaxes with the help of a classic frame-based rectification method [23]. By replacing the conventional feature-matching model with the FD model, the computational complexity of generating putative-correspondences is reduced from $O(N^{2})$ to linear. Based on the FD model, the existing feature-matching algorithms could be applied on matching dense features extracted from high-resolution satellite imagery, even they have high computational complexities.

To ensure that the classic frame-based method is suitable for minimizing the vertical-parallaxes of cross-track pushbroom satellite imagery, a feasibility study was given in this paper by applying the frame-based method on 2.1 million independent experiments which were provided by a cross-track pushbroom based automatic feature-matching evaluating platform [17]. This feasibility study shows that the success rate of the frame-based method for minimizing vertical-parallaxes of cross-track pushbroom images will decline when it is performed on images with large differences of emission angle of the satellite cameras (see Figure 3). In order to increase the success rate, in the FD model, a correspondence-direction-constraint (CDC) algorithm was proposed to get the most favourable seed-matches for the frame-based method. Based on the FD model, the frame-based method could be used for getting reasonable splitting results in all the 2.1 million experiments (see Figure 4).

The procedures of FD model include feature extraction, seed-matches initialization, minimizing vertical-parallaxes, dynamic searching range division and generating putative-correspondences. In the procedure of seed-matches initialization, a hypothesis was presented and verified by 7 million independent experiments for ensuring the initial seed-matches are evenly-distributed, since the evenly-distributed seed-matches can help dynamically adjust the searching ranges in the later procedure. In the procedure of minimizing vertical-parallaxes, the CDC algorithm was proposed for improving the stability of the frame-based method by identifying the most favourable combination of the initial seed-matches based on the k-means method. Eventually, the dynamic searching ranges are generated based on Equations (14)–(16). Because the features in a certain searching range are independent to features in other searching ranges, the matching process can be operated in parallel based on the FD model.

The performances of the FD model and the conventional feature-matching model were evaluated on both the cross-track pushbroom based automatic evaluating platform and real HiRISE images. Three state-of-art feature-matching algorithms (LLT [18], SOCBV [14] and WGTM [13]) were used for evaluating the two models. According to the evaluation results based on the automatic evaluating platform, the running time of the SOCBV and WGTM algorithms reduced substantially when they were based on the FD model. Meanwhile, based on the FD model, all the three algorithms achieved the comparable matching results as they achieved based on the conventional model. On the other hand, the matching results of the two models on real HiRISE images were evaluated manually. The manual evaluating results shows that all the three algorithms achieved extremely good matching results for all the real HiRISE images prepared by this paper.

In the future, the dense and efficient matching results provided by the FD model may be used for automatic epipolar resampling of the cross-track pushbroom satellite imagery. Furthermore, the existing epipolar resampling methods are mostly based on the assumption that the trajectory of the remote sensor is straight [20,21,22]. However, the remote sensors may be suffered high-frequency pointing variations (“jitter”) [29]. Therefore, based on the dense matching results, the assumption of straight trajectory may be eliminated for the future epipolar resampling methods. Eventually, to bring in the best performances of the three feature-matching algorithms, in the experiments of real HiRISE images, the number of correspondences in each local matching was kept just larger than 300, which means the best performance of the FD model was not achieved. Therefore, a more suitable feature-matching algorithm for the FD model could be developed in the future.

## Figures and Tables

**Table 1:** Running time comparison of the three matching algorithms on two feature-matching models with varying numbers of correspondence.

Methods	Number of Simulated Correspondences				
	1000	2000	3000	4000	5000
LLT	0.53 s	0.96 s	1.14 s	1.41 s	**1.83 s**
FD-LLT	0.80 s	1.93 s	1.99 s	2.13 s	3.06 s
SOCBV	1 min 11.27 s	16 min 59.17 s	56 min 47.03 s	2 h 27 min 12.00 s	3 h 4 min 34.68 s
FD-SOCBV	15.38 s	38.15 s	1 min 58.21 s	4 min 10.03 s	**6 min 23.43 s**
WGTM	1 min 57.75 s	21 min 20.23 s	1 h 3 min 39.94 s	2 h 30 min 46.95 s	4 h 24 min 52.70 s
FD-WGTM	10.54 s	17.28 s	57.54 s	2 min 5.13 s	**3 min 4.54**
